# Intake of Blueberry Fermented by *Lactobacillus plantarum* Affects the Gut Microbiota of L-NAME Treated Rats

**DOI:** 10.1155/2013/809128

**Published:** 2013-04-09

**Authors:** Jie Xu, Irini Lazou Ahrén, Olena Prykhodko, Crister Olsson, Siv Ahrné, Göran Molin

**Affiliations:** ^1^Department of Food Technology, Engineering and Nutrition, Lund University, 222 41 Lund, Sweden; ^2^Probi AB, 223 70 Lund, Sweden; ^3^Department of Biology, Lund University, 223 62 Lund, Sweden

## Abstract

Prebiotics, probiotics, or synbiotics can be used as means to regulate the microbiota to exert preventative or beneficial effects to the host. However, not much is known about the effect of the gut microbiota on hypertension which is a major risk factor of cardiovascular disease and also a symptom of the metabolic syndrome. The N^G^-nitro-L-arginine methyl ester (L-NAME) induced hypertensive rats were used in order to test the effect of a synbiotic dietary supplement of *Lactobacillus plantarum* HEAL19 either together with fermented blueberry or with three phenolic compounds synthesized during fermentation. The experimental diets did not lower the blood pressure after 4 weeks. However, the fermented blueberries together with live *L. plantarum* showed protective effect on liver cells indicated by suppressed increase of serum alanine aminotransferase (ALAT) levels. The diversity of the caecal microbiota was neither affected by L-NAME nor the experimental diets. However, inhibition of the nitric oxide synthesis by L-NAME exerted a selection pressure that led to a shift in the bacterial composition. The mixture of fermented blueberries with the bacterial strain altered the caecal microbiota in different direction compared to L-NAME, while the three phenolic compounds together with the bacteria eliminated the selection pressure from the L-NAME.

## 1. Introduction

In recent years, the view of the gut microbiota seen as a metabolic organ has prompted intensive studies on the link between the microbiota and the host health. The plausible roles of the altered microbiota in the development of obesity and type 2 diabetes have been discussed [1, 2]. However, few studies have looked at the relationship between microbiota and hypertension, the latter being an important symptom of the metabolic syndrome and a major risk factor of cardiovascular disease. 

Hypertension characterized as elevated systolic and/or diastolic blood pressure (SBP ≥ 140 mmHg, DBP ≥ 90 mmHg, resp.) is usually treated with antihypertensive agents, but life style modification has also been recommended for both prevention and treatment [3, 4]. One approach to the dietary intervention is to use probiotics together with dietary fibers having prebiotic potential. However the yielding results have so far been inconsistent in improving the hypertensive conditions in both animal models and human trials. In a spontaneously hypertensive rat (SHR) model, administration of milk fermented with *Lactobacillus paracasei *subsp.* paracasei* NTU 101 or *Lactobacillus plantarum* NTU 102 either as a single dose or for 8 weeks, both, significantly decreased SBP and DBP. The authors postulated that the underlying mechanism of the antihypertensive effect could be the result from two substances produced by the two bacteria strains, that is, angiotensin I-converting enzyme inhibitor (ACEI) which is supposed to block the conversion of angiotensin I to angiotensin II and a neurotransmitter *γ*-aminobutyric acid (GABA) [5]. In another study, supplementary tablets made from *Lactobacillus helveticus *CM4 in fermented milk powder were given to people with high-normal blood pressure (SBP = 130–139 mmHg, DBP = 85–89 mmHg) and mild hypertension (SBP = 140–159 mmHg, DBP = 90–99 mmHg) for 4 weeks. A significant decrease in DBP by 5 mmHg but not in SBP was seen in the high-normal blood pressure group, while SBP was significantly decreased by 11.2 mmHg and DBP decreased by 6.5 mmHg (*P* = 0.055) in the mild hypertension group [6]. In contrast, no antihypertensive effect was seen when *Lactobacillus helveticus* Cardio04 in fermented milk was given to human subjects for 8 weeks [7]. 

Dietary flavonoid intake has been shown to have multiple health beneficial effects [8, 9]. Flavonoids, a major group of polyphenols, encompass structurally diverse subclasses which are naturally found in fruits, vegetables, berries, and dark chocolates as well as beverages such as tea and wine. Recent meta-analysis studies confirmed that some of these phenolic compounds such as cocoa flavonols and soy isoflavones were able to reduce blood pressure effectively [10, 11]. Consumption of blueberries (*Vaccinium *spp.), a good source of dietary flavonoids, has also been shown to have beneficial effects on hypertension. Rats fed with a blueberry enriched diet for 6 or 12 weeks showed decreased blood pressure [12]. An antihypertensive effect was also seen in another rat model when the animals were fed blueberry extract for 4 or 6 weeks [13]. Humans with metabolic syndrome who consumed a beverage containing 50 g of freeze dried blueberries for 8 weeks showed a significant decrease in both SBP and DBP [14]. The positive effects were mostly attributed to the antioxidant capacities of anthocyanins. In addition to polyphenols, blueberry also contains essential nutritional components such as vitamin C, folic acid, and minerals, as well as dietary fibers [15].

The antihypertensive effect of using probiotics and blueberries can vary depending on the bacterial strain and blueberry species used and its growth conditions. In the present study we hypothesize that blueberry (*Vaccinium myrtillus*; bilberry in American English) fermented by *Lactobacillus plantarum* HEAL19 (DSM 15313), a tannase producing strain, would exert synbiotic effect on improving the hypertension by modulating the gut microbiota. To test the hypothesis and further elucidate whether the prebiotic effect is from the probiotic fermented whole blueberry or the phenolic compounds found after fermentation, L-NAME induced hypertensive rats were fed with either *L. plantarum* HEAL 19 together with the whole blueberries fermented by the same bacterial strain or with novel phenolic compounds found in the blueberries only after fermentation.

## 2. Materials and Methods

All experiments followed the national guidelines (SFS 1988:534 Swedish Animal Welfare Act, http://www.government.se/content/1/c6/09/03/10/f07ee736.pdf/) for the care and use of animals and were approved by the Malmö/Lund regional ethical committee for laboratory animals (permission number M 83-10).

### 2.1. Animals

Adult male rats (*Rattus norvegicus*) of the Sprague Dawley strain (Mol: SPRD Han, Taconic M & B, Denmark) weighing 200–250 grams (12-13 weeks old) were used in the experiment. The rats were kept in the animal facility at the Department of Biology, Lund University, under pathogen free conditions (20 ± 1°C, 50 ± 10 RH%, 12 : 12 hrs light-dark cycle) in polycarbonate cages on aspen wood bedding material with free access to the water and rodent laboratory chow (product number R34; Lantmännen, Stockholm, Sweden) placed on the lid of cages. Prior to the study, all animals were randomly divided into experimental groups on arrival day with 9 animals per group and kept by three rats per cage during the whole study. Before any experimental procedures, the animals were allowed to acclimatize to their new environment for 7–10 days. At the end of the experiment, the rats were anesthetized with a 3 mL/kg of subcutaneous injection of Hypnorm (fentanyl citrate 0.15 mg/mL; fluanisone 10 mg/mL, Janssen, Oxford, UK) and Dormicum (midazolam, 5 mg/mL, Roche AB, F. Hoffmann-La Roche Ltd. Basel, Switzerland) diluted each in an equal amount of water prior to their mixture in a final ratio of 1 : 1 : 2, respectively.

### 2.2. L-NAME Model

The hypertensive state in the rats was induced by adding the nitric oxide synthase (NOS) inhibitor N^G^-nitro-L-arginine methyl ester (L-NAME) in the drinking water at 400 mg/L. The rats received approximately 40 mg/kg/day of L-NAME for 4 weeks.

### 2.3. Diets/Treatment

Rats were fed standard chow with or without the addition of one of the two study products tested. One study product consisted of blueberries (*Vaccinum myrtillus*) that had been fermented by *Lactobacillus plantarum* HEAL19 (DSM 15313), which is a bacterial strain with a strong tannase activity that can efficiently break down tannins found in blueberries into smaller phenolic acid compounds. The fermented blueberries were freeze dried and milled into a powder. Such powder was given to the rats at 2 g/rat/day (product FBL) mixed with standard chow. The second study product (product PML) consisted of a mixture of three phenolic acids that were absent in the nonfermented blueberries but were detected in the fermented blueberry product. The three phenolic acids were added in the amount that corresponded to their concentration in the daily dosage of product FBL. The phenolic acid mixture consisted of 518 *μ*g of hydroxylactic acid (Sigma, H3253), 2540 *μ*g of phenyllactic acid (Sigma, 113069), and 278 *μ*g of 3,4-dihydroxyphenylpropionic acid (Sigma, 102601). All rats receiving blueberry powder or the phenolic acid mixture were also given 10^9^ cfu/animal/day of *Lactobacillus plantarum* HEAL19 (HEAL19; DSM 15313) by mixing the power with standard chow. The bacterial dose was chosen based on experience from other animal studies with lactobacilli and to reach the amount that could lead to better chance of having positive effect but not too high to make it nonrealistic when converted for possible application to humans. Small boluses were formed by premixing the 15 g of the rodent standard feed in powdered form with 3 mL of water and corresponding product that was recalculated for each rat. The boluses were placed in the “food compartment” on the cage instead of pellets chow for overnight, when rats are most active with eating. Next morning, the pelleted chow was returned, while the rest of the uneaten boluses was removed and recorded. The control group obtained similarly prepared boluses without tested products. Administration of the study products started on the same day as the administration of L-NAME in the drinking water. The consumed amounts of drinking water per cage were recorded every day before administration of fresh water and feed. The following groups of animals were included in the study: (i) control animals not treated with L-NAME receiving standard chow (Ctrl), (ii) L-NAME treated rats receiving standard chow (LN), (iii) L-NAME treated rats receiving blueberry fermented with *L. plantarum* HEAL19 and live HEAL19 (LN + FBL), and (iv) L-NAME treated rats receiving the mixture of three phenolic compounds and live HEAL19 (LN + PML). One animal in the LN + FBL group lost weight and was excluded from data analysis.

### 2.4. Blood-Pressure Measurement

The blood pressure was measured by using the tail-cuff method (CODA, Kent scientific corporations, Torrington, CT, USA) following the manufacturer's instructions. To ensure good circulation of the blood in tail, the animals were kept under heating lamp for the several minutes prior to blood pressure measurement. Then, in quite conditions, the animals were placed into the nose-cone holders on the heating table accompanied the CODA 2 system. After 10 minutes of adaptation to the holders, the cuffs were applied on the tail and 1 run of acclimatization cycle was performed. Totally, 2 sets with 4-5 cycles were performed and result was calculated as a mean per each blood pressure measurement. If animal showed increased heart rate, indicating stress, the blood pressure measurement was repeated later but at the same day. Blood pressure levels were measured at baseline and after 2 and 4 weeks of intervention/treatment. Both systolic (SBP) and diastolic blood pressure (DBP) were measured. Prior to the beginning of study, the animals were trained to the blood pressure measurements to minimize the effect of stress during experimental procedure.

### 2.5. Body Weights

The rats were weighed at baseline and after 2 and 4 weeks of treatment. They were weighed just before having their blood pressure measured.

### 2.6. Blood Sample Analysis

At the end of the experiment, the anesthetized rats were opened and the blood was taken by cardiac puncture into vacutainers for serum. After centrifugations for 10 min at 3000 ×g at 4°C, the serum was collected and stored at −70°C until analysis. Serum alanine aminotransferase (ALAT) was analyzed at the Department of Clinical Chemistry (SUS, Malmö, Sweden) using a COBAS 6000 analyzer (Roche) according to routine methods. 

### 2.7. Caecum Samples

After the opening of the abdomen, the caecal content for microbiological analyses was collected into sterile 2 mL cryovials, immediately placed on dry ice, and kept frozen at −70°C until analyses.

### 2.8. Terminal Restriction Fragment Length Polymorphism (T-RFLP) Analysis

Total DNA was extracted from the caecal content with EZ1 DNA tissue kit (Qiagen AB, Sollentuna, Sweden) on a BioRobot EZ1 workstation (Qiagen). Briefly, approximately 50 mg of the caecal content was suspended in 500 *μ*L of sterile 1x PBS buffer (Oxoid, Basingstoke, UK) and vortexed for 1 min. A sterile 1 *μ*L loop was used to disintegrate big clumps if seen and then 30 min of bead beating step was done on an Eppendorf Mixer (Model 5432, Eppendorf, Hamburg, Germany). Followed by this mechanical lysis, the sample was incubated at room temperature for 10 min, vortexed for 1 min, and centrifuged at 600 ×g for 30 seconds. Then 200 *μ*L of the supernatant was transferred to a sterile sample tube (Qiagen) and total DNA was extracted according to the manufacturer's instructions. The 16S rRNA gene was amplified by using a universal primer pair (Thermo Fisher Scientific, Ulm, Germany) ENV1 (5′-AGAGTTTGATIITGGCTCAG-3′) and ENV2 (5′-CGGITACCTTGTTACGACTT-3′). The 5′ end of ENV1 was fluorescently labelled with FAM dye. The PCR reaction was set up in a total volume of 25 *μ*L consisting of 0.4 *μ*M of FAM-ENV1 primer and 0.2 *μ*M of primer ENV2, 2.5 *μ*L of 10x PCR reaction buffer (500 mM Tris-HCl, 100 mM KCl, 50 mM (NH_4_)_2_SO_4_, 20 mM MgCl_2_, pH 8.3), 0.2 mM of each deoxyribonucleotide triphosphate, 1.25 U of FastStart Taq DNA polymerase (Roche Diagnostics, Mannheim, Germany), and 10–20 ng of template DNA. The PCR was performed under the following condition: 95°C for 3 min, 94°C for 3 min, followed by 30 cycles of 94°C for 1 min, 50°C for 45 s, and 72°C for 2 min. Finally, an additional extension at 72°C for 7 min was done. To decrease the PCR bias, triplicate PCR reactions were prepared for each sample and a negative control was included in every run. The correct amplicon size was checked by agarose gel electrophoresis after each PCR. The resulting PCR products from each sample were pooled together and purified with MinElute PCR purification kit (Qiagen). Then, the DNA concentration was measured by NanoDrop ND-1000 (Saveen Werner, Limhamn, Sweden) and 200 ng of the purified DNA was digested with the endonuclease *Msp*I (Fermentas Life Science, Burlington, Canada) in a total volume of 10 *μ*L according to the manufacturer's instruction. The digested amplicons were analysed on an ABI 3130xl Genetic analyzer (Applied Biosystems, Foster city, CA, USA) with internal size standard GeneScan LIZ 600 (range 20–600 bases, Applied Biosystems) at DNA-lab (SUS, Malmö, Sweden). The resulting T-RFLP data was analyzed using the GeneMapper software version 4.0 (Applied Biosystems) with local southern algorithm. T-RFs were resolved between 40 and 580 bases considering four internal standards were required for accurate sizing of an unknown T-RF. The relative area percentage was calculated for each T-RF which then was used for diversity calculation and principal component analysis (PCA).

### 2.9. SYBR Green Quantitative PCR (qPCR)

Recombinant plasmid standards were constructed by cloning the corresponding 16S rRNA gene fragments specific for the bacterial species into the pGEM-T Vector Systems (Promega, Madison, USA). DNA extracted from bacterial pure cultures was used as follows: *L. plantarum* CCUG 35035 (Culture collection, University of Gothenburg, Sweden) was used to amply the specific region for *Lactobacillus* and total bacteria: *Escherichia coli* CCUG 29300 for *Enterobacteriacea*e, and *Bacteroides fragilis* ATCC 25285 for *B. fragilis *group, and a *Akkermansia* clone obtained from a mouse caecum was used for targeting *Akkermansia muciniphila *specific region on the 16S rRNA gene. The bacterial groups were amplified using QuantiTect SYBR Green PCR kit (Qiagen) in a real-time PCR cycler Rotor-Gene Q (Qiagen). The primers and annealing temperatures used in the study are listed in Table 1. The PCR profile was set as follows: activation at 95°C for 15 min followed by 40 cycles of (1) 95°C for 15 sec, (2) annealing at 50–60°C for 30–60 sec, and (3) extension at 72°C for 30 or 60 sec. Melt curve analysis was performed for each run to check the specificity of the primers. The PCR reaction was prepared in 20 *μ*L consisting 10 *μ*L of 2x QuantiTect SYBR Green PCR Master mix, 0.5 *μ*M of each primer, and 2 *μ*L of template DNA. If necessary, template DNA was diluted 10–100-fold prior to qPCR. Triplicate reactions were performed for each sample, standard, and negative controls.

### 2.10. Statistics

The blood pressure and body weight data were normally distributed and analysed with one-way ANOVA and a Tukey HSD post hoc test for pairwise comparison if necessary using package “stats” in the R (version 2.15.1) program. The values are presented as the mean ± SE. Values of *P* < 0.05 were considered statistically significant.

The ALAT enzyme, Shannon's diversity index [21], and qPCR data were not normally distributed and thus evaluated with nonparametric test Kruskal-Wallis test and Nemenyi-Damico-Wolfe-Dunn test (NDWD) for pairwise comparisons using package “coin” in the R program. Values of *P* < 0.05 were considered statistically significant.

T-RFLP data was analyzed with principal component analysis (PCA) with SIMCA-P software (version 12.0.1.0; Umetrics, Umeå, Sweden).

## 3. Results

### 3.1. Normal Growth of Animals in All Groups

The experimental diets did not affect the normal growth of the animals. There was no significant difference in body weight gain after 4 weeks between groups (76.1 ± 5.0 g, 85.1 ± 4.4 g, 83.4 ± 5.6 g, 83.3 ± 8.5 g for Ctrl, LN, LN + FBL, and LN + PML, resp.).

### 3.2. Experimental Diets Had No Effect on Blood Pressure

**Figure 1:**
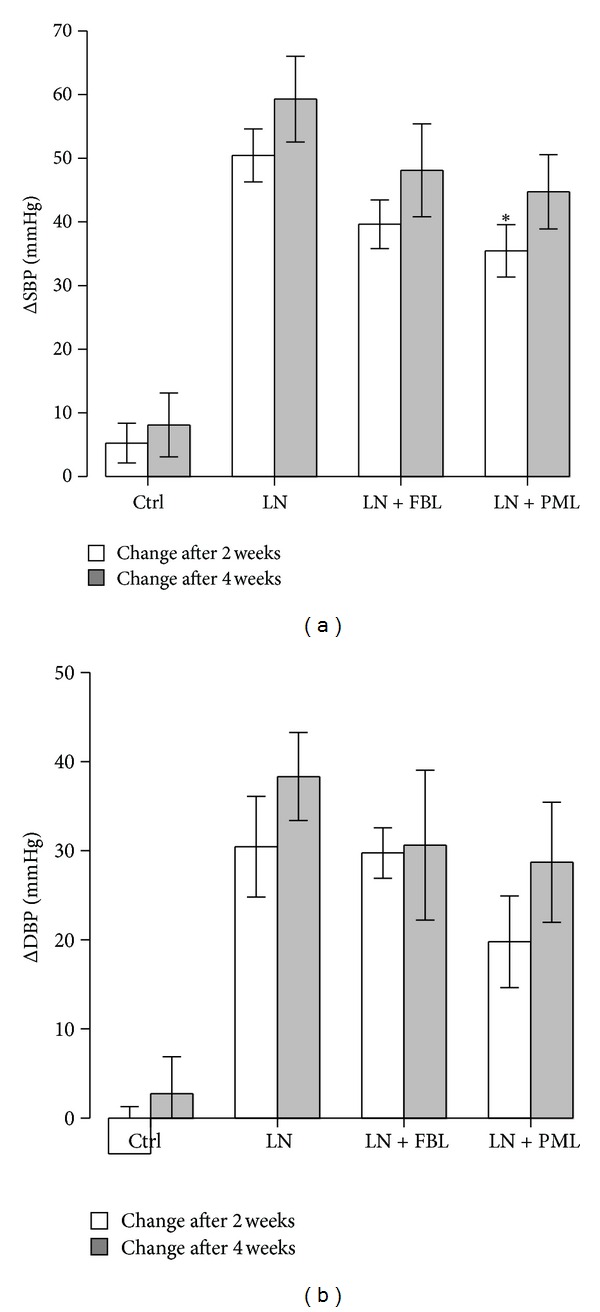
Blood pressure changes after 2 and 4 weeks treatment. (a) Changes in SBP. (b) Changes in DBP. Ctrl: control (*n* = 9); LN: group receiving standard chow and L-NAME in drinking water (*n* = 9); LN + FBL: group receiving L-NAME in drinking water and fermented bluberry + *L. plantarum *HEAL19 added to feed (*n* = 8); LN + PML: group receiving L-NAME in drinking water and three phenolic acid mixture + *L. plantarum *HEAL19 added to feed (*n* = 9). Both SBP and DBP significantly increased in L-NAME treated groups after 2 and 4 weeks when compared to the Ctrl. Data are means ± SE. *P < 0.05 compared to LN.

The L-NAME treatment significantly elevated both SBP (*P* < 0.001) and DBP (*P* < 0.001) after 2 weeks. The significant increase in BP remained after 4 weeks (*P* < 0.001 for SBP and *P* < 0.01 for DBP). There was a reduction in SBP after 2 weeks in LN + PML group (*P* = 0.04) compared to LN. However, the experimental diets had no effect either on SBP or DBP after 4-week treatment (Figure 1).

### 3.3. LN + FBL Did Not Increase Liver Enzyme ALAT

**Figure 2:**
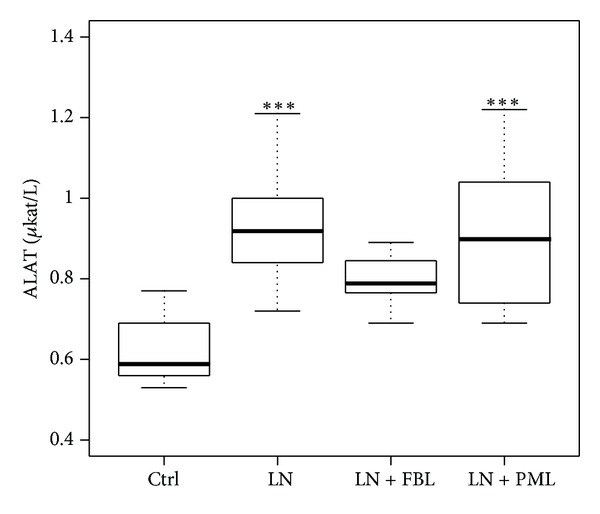
Serum ALAT enzyme. Ctrl: control (*n* = 9); LN: group receiving standard chow and L-NAME in drinking water (*n* = 9); LN + FBL: group receiving L-NAME in drinking water and fermented bluberry + *L. plantarum *HEAL19 added to feed (*n* = 8); LN + PML: group receiving L-NAME and three phenolic acid mixture + *L. plantarum *HEAL19 (*n* = 9). ***P < 0.001 compared to Ctrl.

The serum ALAT levels were significantly increased in LN (*P* < 0.001) and LN + PML (*P* < 0.001) groups compared to Ctrl (Figure 2). However, no statistically significant increase was detected in LN + FBL (*P* = 0.06). 

### 3.4. No Change in Microbial Diversity after Inducing Hypertension

Shannon's diversity index was used to compare the microbial diversities. Neither the L-NAME treatment nor the experimental diets did change the caecal microbial diversity (Table 2). 

### 3.5. Certain Bacterial Population Altered after Experimental Diet in Rats with Hypertension

qPCR was performed to see whether the induction of hypertension or the experimental diets would alter the bacterial composition in the caecum content (Table 3). Induction of hypertension by L-NAME did not significantly change any of the tested bacterial population. Even though not statistically significant, there was a trend of increase in *Enterobacteriaceae* population in LN group (*P* = 0.064). The supplementation of the FBL significantly decreased the *Lactobacillus *(*P* = 0.023) and *B. fragilis* group (*P* = 0.016) populations compared to LN but not to Ctrl. The addition of the PML showed a trend of increase in *Akkermansia* population compared to LN group (*P* = 0.051). The expected increase in *Lactobacillus *was not seen either in LN + FBL or LN + PML group; this could be due to the unsuccessful colonization of the *L. plantarum* HEAL19.

### 3.6. Microbiota Compositional Change after Hypertension Induction

**Figure 3:**
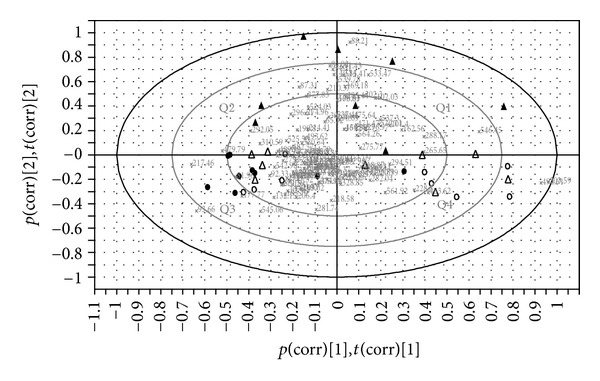
PCA loadings biplot. Open circle (Ctrl): control rats (*n* = 9); dots (LN): rats receiving standard chow and L-NAME in drinking water (*n* = 9); triangles (LN + FBL): rats receiving L-NAME in drinking water and fermented bluberry + *L. plantarum *HEAL19 added to feed (*n* = 8); open triangles (LN + PML): rats receiving L-NAME in drinking water and three phenolic acid mixture + *L. plantarum *HEAL19 added to feed (*n* = 9). The numeric numbers indicate the sizes in bases of the detected T-RFs representing different bacterial groups. Q1–Q4 denote 1st to 4th quadrants.

The PCA analysis was applied on the T-RFLP data to illustrate the variation of the caecal microbiota between the groups. The data matrix composed of relative area of the T-RFs was not scaled but centered and the first two principal components (PCs) were calculated. PC1 explained 31.1% of the variance and PC2 explained 17.7%. PCA loadings biplot (Figure 3) showed the correlation between T-RFs representing different bacteria groups and also the similarity of the microbiota between the individual animals based on the T-RF distribution. LN + FBL group was separated from other groups mostly by PC2 and showed large individual variation scattered in the first and second quadrants. In addition, bacteria groups represented by T-RF88 and T-RF533 were most abundant in LN + FBL group. Animals from LN group were found mostly in the third quadrant except for one rat was found in the fourth quadrant, suggesting that they were sharing more homogeneous microbiota. LN group harbored high abundance of T-RF93 and T-RF217 but suppressed bacteria group represented by T-RF303. Animals from Ctrl and LN + PML groups were mostly located in the third and fourth quadrants, indicating that the LN + PML combination did not affect the caecal microbiota.

## 4. Discussion

None of the experimental diets had a blood pressure lowering effect, neither on SBP nor on DBP after 4 weeks. However, the experimental diet FBL which is the supplementation of *L. plantarum *HEAL19 with blueberries fermented by the same bacterial strain reduced the liver cell damage as observed by suppressed increase of serum ALAT level. ALAT, an enzyme mainly found in cytosolic side of liver cells, would leak into the blood stream if the liver cell is damaged. Elevated serum ALAT level has been reported to be associated with various liver diseases [22, 23]. L-NAME treatment significantly increased the ALAT level in this study. However, experimental diet FBL seemed to counteract the adverse effect of L-NAME on the ALAT increase, suggesting a possible protecting effect on hepatocytes. Consumption of blueberries has been reported to reduce hepatocyte injury, lipid peroxidation [24], and oxidative stress [25, 26]. Furthermore, fermentation of blueberries has been shown to increase the total polyphenols and antioxidant capacities [27, 28]. Thus, the fermentation of blueberries may have enhanced the scavenging of free radicals that may antagonize the development of liver injury. 

Nitric oxide, a hydrophobic signaling molecule which is small enough to pass plasma membranes freely, is known to be involved in various physiological functions including antimicrobial activity [29, 30]. L-NAME that blocks the NO production has been shown to also decrease oxygen delivery to the gut [31]. Thus, it is plausible to assume that inhibition of the nitric oxide synthesis by L-NAME could have an effect on the oxygen tension in the microenvironment close to the intestinal mucosa, which might favor some components of the microbiota and disfavor others. In the present study, a shift was seen in the composition of the microbiota in the LN group by PCA analysis. L-NAME treatment has been reported to increase the viable count of *Enterobacteriaceae* in the gut of rats [32]. Although not statistically significant, a trend of increase in *Enterobacteriaceae* after L-NAME treatment was seen in this study but the supplementation of the FBL or PML seemed suppressed the growth of the *Enterobacteriaceae* (Table 3). This could be attributed to the antimicrobial effects from the experimental diets. In addition, L-NAME treatment also increased the total bacterial load in the caecum and colon in rats with liver injury [33]. However, we did not find significant differences in total bacteria load measured by qPCR in the present model which might be partly explained by the difference between culture-dependent and independent methods that were used. 

L-NAME treatment has been reported to prevent the bacterial translocation by protecting the integrity of the GI tract in hemorrhagic shocked animals [34], while in healthy rats, administration of L-NAME increased the intestinal motility and the load of mucosa associated bacteria and the effects were suggested to be a consequence of a decreased mucus secretion [35]. In the present study, the amount of *Akkermansia* that are mucin degrading bacteria did not decrease after L-NAME treatment, which implies that mucus secretion has been unaffected in the present model, at least in the caecum. However, further studies on mucin gene expressions and quantification of the caecal mucin content are needed to confirm such a relationship.

The addition of the *L. plantarum *HEAL19 did not result in an expected increase of *Lactobacillus *population either in LN + FBL or LN + PML group. The T-RF568 that was putatively identified as the *L. plantarum* HEAL19 in previous work [36] has been only detected in one rat from the LN + FBL with low abundance around 0.5%. Thus it can be speculated that the survival rate of the bacterial strain was low. Antimicrobial activities of different blueberry species were observed to selectively inhibit growth of pathogens when tested as whole berry, berry fractions, extracts of phenolic compounds, and ethanol extract [37–39]. The significantly decreased population of *Lactobacillus* and *B. fragilis* group in the LN + FBL group as compared with LN group could be a result from such selection pressure of the blueberries. Despite the effects of the diets observed on the certain group of bacteria measured by qPCR, an overview of the caecal microbiota composition by PCA analysis revealed that large variations exist among the individuals upon the response to treatments. One contributing factor could be the different individual's original microbiota.

Interestingly, the phenolic compounds together with *L. plantarum* HEAL19 did not affect the composition of the microbiota as shown by PCA analysis (Figure 3). The selection pressure from either L-NAME or phenolic compounds seemed to be eliminated. Phenyllactic acid (PLA) and hydroxyphenyllactic acid (HPLA), metabolites of phenylalanine and tyrosine, respectively, have previously been shown to have antibacterial and antifungal activities and can also be produced by *Lactobacillus plantarum* stains [40–42]. In the present study no such effect was observed on the caecal microbiota. It can be speculated that the compounds did not reach the effective concentration in the caecum. Another phenolic compound included in the experimental diet 3,4-dihydroxyhydrocinnamic acid (DHCA) is a metabolite of caffeic acid. A recent study has shown that DHCA enhanced the activity of endothelial nitric oxide synthase (eNOS) *in vitro* [43]. This provides a possible reason for the observation in PCA analysis that shows that the L-NAME effect on the microbiota was eliminated by the PML diet.

In conclusion, the main findings of this study are that (1) L-NAME alone conferred selective pressure on the caecal microbiota, (2) L-NAME with blueberries fermented with *L. plantarum* HEAL19 showed protective effect against liver cell damage and altered the caecal microbiota to different degrees in different individuals, and (3) the combination of the three phenolic compounds found in blueberries fermented with *L. plantarum* HEAL19 eliminated selection pressure on the caecal microbiota induced by L-NAME. 

## Figures and Tables

**Table 1:** Primers and annealing conditions used in qPCR for bacterial quantification.

Name	Sequence (5′-3′)	Target group	Amplicon size (bp)	Annealing temperature (°C)	Reference
Lact-16S-F Lact-16S-R	GGAATCTTCCACAATGGACG CGCTTTACGCCCAATAAATCCGG	*Lactobacillus *	217	56	[16]
Eco1457-F Eco1652-R	CATTGACGTTACCCGCAGAAGAAGC CTCTACGAGACTCAAGCTTGC	*Enterobacteriaceae *	195	60	[17]
g-Bfra-F g-Bfra-R	ATAGCCTTTCGAAAGRAAGAT CCAGTATCAACTGCAATTTTA	*Bacteroides fragilis* group	495	50	[18]
AM1-F AM2-R	CAGCACGTGAAGGTGGGGAC CCTTGCGGTTGGCTTCAGAT	*Akkermansia *	327	60	[19]
Tot-F Tot-R	GCAGGCCTAACACATGCAAGTC CTGCTGCCTCCCGTAGGAGT	Total bacteria	292	60	[20]

**Table 2:** Caecal microbiota diversity indices.

	Shannon's diversity indices	
	Median	25–75%
Ctrl	2.90	2.82–2.99
LN	2.96	2.85–2.98
LN + FBL	2.72	2.55–2.88
LN + PML	2.97	2.82–2.99

Ctrl: control (*n* = 9); LN: group receiving standard chow and L-NAME in drinking water (*n* = 9); LN + FBL: group receiving L-NAME in drinking water and fermented bluberry + *L. plantarum* HEAL19 added to feed (*n* = 8); LN + PML: group receiving L-NAME and three phenolic acid mixture + *L. plantarum* HEAL19 (*n* = 9). Data are expressed as median values and 25–75 percentiles.

**Table 3:** SYBR Green qPCR of bacterial 16S rDNA in rat caecal content.

	Ctrl		LN		LN + FBL		LN + PML	
	Median*	25–75%	Median	25–75%	Median	25–75%	Median	25–75%
*Lactobacillus *	8.35^ab^	7.93–8.77	8.54^b^	8.50–8.86	7.84^a^	7.67–8.16	8.96^b^	8.71–9.07
*Enterobacteriaceae*	7.64	7.39–7.77	8.07	7.83–8.14	7.14	6.64–7.76	7.64	7.43–7.95
*Bacteroides fragilis* group	11.26^ab^	11.19–11.64	11.82^b^	11.13–11.99	9.2^a^	7.45–11.00	11.18^ab^	10.86–11.86
*Akkermansia *	8.63	8.00–9.15	8.60	8.09–8.89	8.56	8.14–9.06	9.23	9.17–9.50
Total bacteria	10.17	10.05–10.42	10.03	9.61–10.60	9.97	9.72–10.18	10.18	9.79–10.23

Ctrl: control (*n* = 9); LN: group receiving standard chow and L-NAME in drinking water (*n* = 9); LN + FBL: group receiving L-NAME in drinking water and fermented blueberry + *L. plantarum* HEAL19 added to feed (*n* = 8); LN + PML: group receiving L-NAME and three phenolic acid mixture + *L. plantarum* HEAL19 (*n* = 9). Data are expressed as median values and 25–75 percentiles. Groups marked with superscript letters that do not share the same letter were significantly different (*P* < 0.05). *Data are expressed as median log copy/g and 25–75 percentiles.
